# Normosol-R vs Lactated Ringers in the Critically Ill: A Randomized Trial

**DOI:** 10.1016/j.chest.2025.02.008

**Published:** 2025-02-17

**Authors:** Edward T. Qian, Ryan M. Brown, Karen E. Jackson, Li Wang, Joanna L. Stollings, Robert E. Freundlich, Jonathan P. Wanderer, Edward D. Siew, Gordon R. Bernard, Wesley H. Self, Jonathan D. Casey, Todd W. Rice, Matthew W. Semler

**Affiliations:** a Department of Medicine, Division of Allergy, Pulmonary and Critical Care Medicine, Nashville, TN; b Department of Anesthesiology, Vanderbilt University Medical Center, Nashville, TN; c Department of Biomedical Informatics, Vanderbilt University Medical Center, Nashville, TN; d Asheville Pulmonary and Critical Care Associates, Asheville, NC; e Division of Pulmonary, Critical Care, and Sleep Medicine, Rush University Medical Center, Chicago, IL; f Department of Biostatistics, Vanderbilt University, Nashville, TN; g Department of Pharmaceutical Services, Vanderbilt University Medical Center, Nashville, TN; h Critical Illness, Brain Dysfunction, and Survivorship Center, Vanderbilt University Medical Center, Nashville, TN; i Department of Medicine, Division of Nephrology and Hypertension, Vanderbilt University Medical Center, Tennessee Valley Health Systems (TVHS), Nashville Veterans Affairs Hospital, Nashville, TN; j Vanderbilt Institute for Clinical and Translational Research (VICTR), Vanderbilt University Medical Center, Nashville, TN; k Department of Emergency Medicine, Vanderbilt University Medical Center, Nashville, TN

**Keywords:** balanced crystalloid, bicarbonate, critical illness

## Abstract

**Background:**

Balanced crystalloid solutions may improve clinical outcomes compared with saline for some critically ill adults, but it is unclear whether differences in composition between balanced crystalloid solutions affect outcomes.

**Research Question:**

Does the choice of balanced crystalloid solutions (Normosol-R vs lactated Ringers) impact acid-base status, organ function, or outcomes for critically ill adults?

**Study Design and Methods:**

This pragmatic, cluster-randomized, multiple-crossover trial at an academic medical center in the United States compared the use of Normosol-R vs lactated Ringers among critically ill adults. The primary outcome was the plasma bicarbonate (HCO_3_^−^) concentration between enrollment and 7 days. Secondary outcomes included receipt of kidney replacement therapy and death by day 30.

**Results:**

Between June 1, 2018, and January 31, 2019, 2,084 patients were enrolled. The median age was 59 years, 1,006 patients (48.3%) were female, and the median Sequential Organ Failure Assessment score was 5 (interquartile range, 3-8). HCO_3_^−^ concentration between enrollment and 7 days did not differ between the Normosol-R and lactated Ringers groups (mean difference, −0.12 mmol/dL; 95% CI, −0.61 to 0.36; *P* = .61). A total of 58 patients (6.0%) in the Normosol-R group and 47 patients (5.0%) in the lactated Ringers group received new kidney replacement therapy (absolute risk difference, 1.0%; 95% CI, −1.2% to 3.1%). Death by day 30 occurred in 172 patients (16.3%) in the Normosol-R group and 164 patients (16.0%) in the lactated Ringers group (absolute risk difference, 0.3%; 95% CI, −2.9% to 3.6%).

**Interpretation:**

Among critically ill adults, the use of Normosol-R for IV fluid therapy did not affect plasma HCO_3_^−^ concentrations or clinical outcomes compared with lactated Ringers.

**Clinical Trial Registration:**

ClinicalTrials.gov; No.: NCT03537898; URL: www.clinicaltrials.gov

## Graphical Abstract

**Figure:**
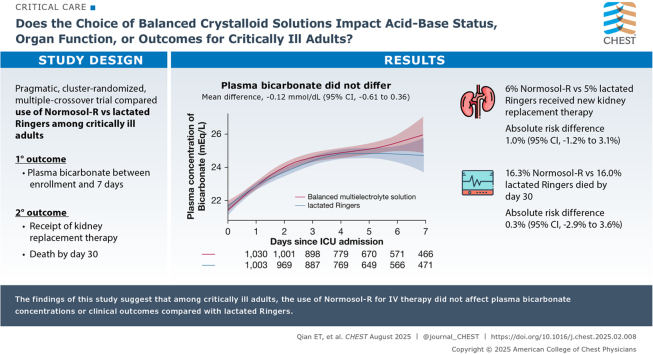

**Take-Home Points**

**Study Question:** In critically ill adults received IV crystalloid, does the choice of balanced crystalloid solution between Normosol-R and lactated Ringers affect bicarbonate (HCO_3_^−^) concentration or other clinical outcomes?

**Results:** HCO_3_^−^ concentration between enrollment and 7 days did not differ between the Normosol-R and lactated Ringers groups (mean difference, −0.12 mmol/dL; 95% CI, −0.61 to 0.36; *P* = .61). A total of 58 patients (6.0%) in the Normosol-R group and 47 patients (5.0%) in the lactated Ringers group received new kidney replacement therapy (absolute risk difference, 1.0%; 95% CI, −1.2% to 3.1%). Death by day 30 occurred in 172 patients (16.3%) in the Normosol-R group and 164 patients (16.0%) in the lactated Ringers group (absolute risk difference, 0.3%; 95% CI, −2.9% to 3.6%).

**Interpretation:** Among critically ill adults, the use of Normosol-R for IV fluid therapy did not affect plasma HCO_3_^−^ concentrations or clinical outcomes compared with lactated Ringers.

The IV administration of crystalloid solutions is common in the ICU.^1^^,^^2^ Randomized trials and meta-analyses have reported a high probability that using balanced crystalloid solutions reduces mortality for critically ill adults compared with saline.^3^^,^^4^ Although some prior research has considered all balanced crystalloid solutions as a single group, the balanced crystalloid solutions that are available and commonly used in current clinical care differ significantly in their composition in ways that may affect acid-base balance, organ function, and outcomes.

Two common types of balanced crystalloid solution include balanced multielectrolyte solutions buffered with acetate, gluconate, or both (eg, Normosol-R, Plasmalyte-A) and lactate-buffered solutions (eg, Hartmann’s solutions, lactated Ringers). Normosol-R and Plasmalyte-A differ from lactated Ringers in their concentrations of sodium (140 vs 130 mM), chloride (98 vs 109 mM), lactate (0 vs 28 mM), acetate (27 vs 0 mM), and gluconate (23 vs 0 mM). The resulting difference between the fluids in the strong ion difference (50 vs 28 mM) has been hypothesized to affect the acid-base balance in patients receiving IV fluid therapy.^5^ Although hyperchloremia and nonanion gap metabolic acidosis have been proposed to mediate the relationship between IV fluid composition and kidney replacement therapy or death in critically ill adults, to our knowledge, whether the choice of balanced crystalloid solution affects acid-base status, organ function, or death for critically ill adults has never been evaluated in a randomized trial.^6^, ^7^, ^8^ This difference is important to clinicians, and 87% of respondents to a recent international survey of anesthesiologists and intensivists support a randomized trial evaluating acetate vs lactate buffered solutions.^9^

To determine the effect of balanced crystalloid solution composition on plasma laboratory values, organ function, and clinical outcomes in critically ill adults, we conducted the Balanced Solutions and Plasma Electrolytes (BASE) trial. We hypothesized that use of Normosol-R would result in higher plasma bicarbonate (HCO_3_^−^) concentrations compared with use of lactated Ringers.

## Study Design and Methods

### Study Design and Oversight

We conducted a pragmatic, unmasked, single-center, cluster-randomized, multiple-crossover trial comparing the use of a balanced crystalloid solution buffered with acetate and gluconate (Normosol-R) and a balanced crystalloid solution buffered with lactate (lactated Ringers) among critically ill adults admitted to the medical ICU at an academic medical center in the United States. The trial was approved by the institutional review board at Vanderbilt University Medical Center with a waiver of informed consent (e-Appendix 1) and was registered online before initiation (NCT03537898). The statistical analysis plan was finalized before completion of the trial.

### Patient Population

All adults (≥ 18 years of age) admitted to the medical ICU during the trial were enrolled at the time of ICU admission. Enrolled patients who were discharged from the hospital were eligible to participate again if they were readmitted to the medical ICU during the trial period.

### Randomization

Each month of the trial, the participating ICU was assigned to use either Normosol-R or lactated Ringers. Using a single computer-generated simple randomization, the ICU was assigned to use Normosol-R during even months and lactated Ringers during odd months. During the 8 months of the trial, the ICU alternated the assigned fluid each month, for a total of 4 months assigned to Normosol-R and 4 months assigned to lactated Ringers. Patients, clinicians, and investigators were not masked to group assignment. During the trial period, the use of balanced crystalloids in the emergency department was coordinated with the fluid assigned to the participating ICU such that, whenever feasible, patients for whom admission to the ICU was planned received the same balanced crystalloid in the emergency department that was assigned to the ICU for that month.

### Treatments

Patients in the Normosol-R group were assigned to receive Normosol-R whenever IV crystalloid was administered, and patients in the lactated Ringers group were assigned to receive lactated Ringers whenever IV crystalloid was administered. This included fluid boluses, fluid infusions (ie, maintenance fluids), medication carriers, and minimum infusion rates (keep vein open) but did not include medication diluents. Whenever clinicians initiated an order for an isotonic crystalloid solution (eg, Normosol-R, lactated Ringers, 0.9% sodium chloride), an electronic advisor in the order-entry system informed providers about the trial, asked about relative contraindications to the assigned balanced crystalloid, and if none were present, guided providers to order the assigned balanced crystalloid. For patients with an absolute or relative contraindication to the use of balanced crystalloid solutions (eg, traumatic brain injury), the use of 0.9% sodium chloride was permitted. If clinicians thought it to be required for the optimal treatment of any patient, the nonassigned balanced crystalloid was also available from the pharmacy.

### Data Collection

We used data collected in routine care and electronically extracted from electronic health records.^7^^,^^10^^,^^11^ These data included information on preenrollment demographic characteristics, admitting location and diagnosis, kidney function, and severity of illness; receipt of IV crystalloids, other fluids, and blood products; serum electrolyte and creatinine values; receipt of kidney replacement therapy, mechanical ventilation, and vasopressors; and vital status and serum creatinine at hospital discharge. Trial personnel performed manual chart review to identify indications for new kidney replacement therapy.

### Study Outcomes

The primary outcome was the plasma HCO_3_^−^ concentration over the first 7 days after enrollment. Secondary laboratory outcomes included plasma concentrations of sodium, potassium, chloride, HCO_3_^−^ , blood urea nitrogen, creatinine, calcium, and lactate from enrollment through hospital discharge or 30 days (e-Appendix 1). Secondary outcomes related to acid-base status included arterial pH, arterial standard base excess, and strong ion difference, calculated as (sodium + potassium + calcium) – (chloride + lactate). The strong ion difference is equal to the concentration of buffer base in the serum, where larger values suggest the presence of more weak acid in the serum and smaller values suggest the presence of less weak acid.^12^

Secondary clinical outcomes included 30-day in-hospital mortality, receipt of new kidney replacement therapy, persistent renal dysfunction, and Major Adverse Kidney Events within the 30 days after enrollment—defined as the composite of in-hospital mortality, receipt of new kidney replacement therapy, or persistent renal dysfunction (a final inpatient serum creatinine value ≥ 200% of baseline). Additional outcomes included the following: stage II or higher acute kidney injury by Kidney Disease Improving Global Outcomes (KDIGO) creatinine criteria^13^ between enrollment and 7 days; dose of vasopressors between enrollment and 7 days; and ICU-free days, ventilator free-days, vasopressor-free days, and kidney replacement therapy-free days at 28 days after enrollment (e-Appendix 1). For evaluating outcomes related to kidney injury, the value for baseline serum creatinine was determined using a previously described hierarchical approach (e-Appendix 1).^10^ Patients who had received kidney replacement therapy before enrollment were considered ineligible to experience acute kidney injury, new kidney replacement therapy, or persistent renal dysfunction.

### Statistical Analysis

The sample size estimation for this trial was based on data from prior cluster-crossover trials in the same setting comparing balanced crystalloids with saline. In those trials, the enrollment rate was approximately 250 patients per month, the mean plasma HCO_3_^−^ concentration between enrollment and 7 days ± SD was 24.0 ± 6.0 mM, and the difference between groups in plasma HCO_3_^−^ concentration was approximately 1.0 mM on days 1 through 7 after enrollment.^7^ Using these data, we calculated that achieving 90% statistical power at a 2-sided alpha level of .05 to detect a difference in plasma HCO_3_^−^ concentration of 0.9 mM between groups would require enrollment of at least 1,800 patients. To ensure enrollment of at least 1,800 patients and an equal number of months assigned to each of the 2 trial groups, we planned to enroll for a total of 8 months.

Analyses were conducted at the level of each patient’s hospitalization in an intention-to-treat fashion. Continuous variables are reported as means and SDs or as medians and interquartile ranges (IQRs); categorical variables are reported as frequencies and percentages.

The primary analysis was an intention-to-treat comparison of the primary outcome of plasma HCO_3_^−^ concentration from enrollment to 7 days between the Normosol-R group and the lactated Ringers group. Because patients could have multiple HCO_3_^−^ measurements during the outcome period and these values were not independent of one another because they occurred in an individual patient, a linear mixed-effects model was used with a random effect for patient within period to account for correlation within patients and within study months and the following prespecified baseline covariates: age, sex, race, source of admission to the ICU, receipt of mechanical ventilation, receipt of vasopressors, diagnosis of sepsis, and diagnosis of cirrhosis. These variables were adapted from a prior randomized trial of IV crystalloid in critically ill adults removing trial site because our trial was performed in a single ICU and adding cirrhosis because patients with cirrhosis have disordered lactate metabolism.^7^

In sensitivity analyses, we excluded patients enrolled the 7 days before a crossover in the assigned fluid to simulate a washout period, and included only each patient’s first admission.

To assess for effect modification, we fit a linear mixed-effects model for the primary outcome of plasma HCO_3_^−^ concentration or a generalized linear mixed-effects model for the secondary outcome of 30-day in-hospital mortality with the same baseline covariates prespecified for plasma HCO_3_^−^ concentration and an interaction term between trial group assignment and the following prespecified baseline variables: baseline plasma HCO_3_^−^ concentration, source of admission to the ICU, diagnosis of sepsis, diagnosis of cirrhosis, receipt of mechanical ventilation, receipt of vasopressors, category of kidney dysfunction, and baseline Sequential Organ Failure Assessment score.^14^ Using a similar approach, we evaluated whether the volume of fluid received between enrollment and day 30 modified the effect of trial group assignment on outcomes.

Additional analyses compared secondary outcomes between trial groups and evaluated for a correlation between measured strong ion difference and plasma HCO_3_^−^.

A 2-sided *P* < .05 indicated statistical significance for the primary outcome. Between-group differences in secondary outcomes are reported as point estimates and 95% CIs. The widths of the CIs were not adjusted for multiplicity and should not be used to infer definitive differences in treatment effects between the 2 groups. When data were missing for primary, secondary, or exploratory outcomes, complete case analysis was performed; no data on outcomes were imputed. In adjusted analyses, missing data for baseline covariates were imputed using multiple imputations. All the analyses were performed with the use of R software, version 4.3.0 (R Foundation for Statistical Computing).

### Role of the Funding Source

The funders of the study had no role in study design, data collection, data analysis, data interpretation, or writing of the report.

## Results

**Figure 1:**
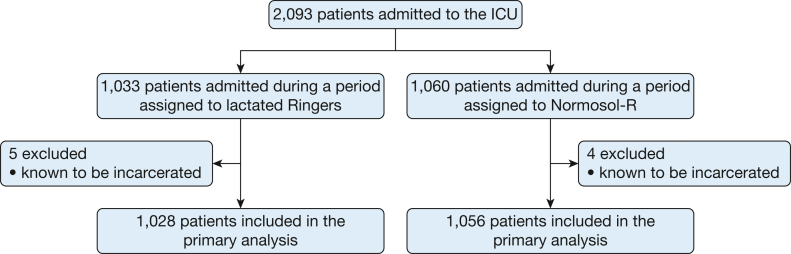
Consolidated Standards of Reporting Trials diagram. Of 2,093 patients admitted to the ICU during the study period, 9 were known to be incarcerated and were excluded, and 2,084 patients were included in the primary analysis.

Between June 1, 2018, and January 31, 2019, a total of 2,084 patients were enrolled in the trial (Fig 1). The median age was 58.9 years, and 1,006 patients (48.3%) were female. At the time of enrollment, 544 patients (26.1%) were receiving mechanical ventilation and 465 patients (22.3%) were receiving vasopressors. The baseline characteristics of the 1,056 patients (50.7%) assigned to the Normosol-R group and the 1,028 patients (49.3%) assigned to the lactated Ringers group were similar (e-Table 1, Table 1).

**Table 1:** Patient Characteristics at Baseline

Patient Characteristics	Normosol-R	Lactated Ringers
	(n = 1,056)	(n = 1,028)
Age, y	58.5 (43.6-68.0)	59.5 (46.2-68.8)
Male	550 (52.1)	528 (51.4)
Race and ethnicity		
Black, non-Hispanic	222 (21.0)	192 (18.7)
Hispanic	19 (1.8)	18 (1.8)
Not reported	22 (2.1)	21 (2.0)
Other^a^	32 (3.0)	34 (3.3)
White, non-Hispanic	761 (72.1)	763 (74.2)
Height, cm^b^	170.2 (162.6-177.8)	170.2 (162.6-177.8)
Weight, kg^c^	78.9 (64.7-95.5)	77.6 (62.6-96.6)
Chronic comorbidities		
Pulmonary^d^	272/905 (30.1)	309/886 (34.9)
Chronic heart failure^d^	261/905 (28.8)	277/886 (31.3)
Cirrhosis	68/1,026 (6.6)	68/1,005 (6.8)
Prior kidney replacement therapy receipt	82 (7.8)	86 (8.4)
Acute kidney injury, stage II or greater^e^	324/974 (33.3)	314/942 (33.3)
Sepsis as primary diagnosis at ICU admission^f^	151/1,026 (14.7)	163/1,005 (16.2)
SOFA score^g^	5.0 (3.0-8.0)	5.0 (3.0-8.0)
Vasopressors	234 (22.2)	231 (22.5)
Mechanical ventilation	272 (25.8)	272 (26.5)
Plasma laboratory values at enrollment		
HCO_3_^−^, mM	22.0 (18.0-25.0)	22.0 (18.0-25.0)
Chloride, mM	104.0 (99.0-108.0)	104.0 (99.0-108.0)
Creatinine, mg/dL^h^	1.2 (0.8-2.3)	1.2 (0.8-2.3)
Source of admission to the ICU		
Emergency department	615 (58.2)	645 (62.7)
Transfer from another hospital	177 (16.8)	141 (13.7)
Hospital ward	179 (17.0)	188 (18.3)
Operating room	22 (2.1)	17 (1.7)
Another ICU within the hospital	55 (5.2)	32 (3.1)
Outpatient	8 (0.8)	5 (0.5)

Continuous data are presented as median (25th percentile-75th percentile) or mean (SD), and categorical data are presented as No. (%) or No./total No. (%). There were no significant differences in baseline characteristics between the 2 study groups (*P* values range, .20-.97). HCO_3_^−^ = bicarbonate; SOFA = Sequential Organ Failure Assessment.

a Other includes the following: American Indian or Alaska Native, Asian Indian, Chinese, decline to answer, other Asian, other Pacific Islander, or unknown.

b Information on weight at ICU admission was missing for 24 patients (11 in the lactated Ringers group and 13 in the Normosol-R group).

c Information on height was missing for 206 patients (105 in the lactated Ringers group and 101 in the Normosol-R group).

d Defined by the Charlson Comorbidity Index.

e Acute kidney injury stage II or greater is defined according to Kidney Disease Improving Global Outcomes creatinine criteria^13^ as a first measured plasma creatinine value after ICU admission of at least 200% of the baseline value or both (1) > 4.0 mg/dL and (2) increased at least 0.3 mg/dL from the baseline value. Acute kidney injury only reported in the 1,916 patients without prior kidney replacement therapy.

f Sepsis-Related Organ Failure.

g SOFA score, also known as the Sepsis-related Organ Failure Assessment score,^14^ was calculated using data collected from the day of ICU admission.

h Baseline creatinine for the study was defined as the lowest plasma creatinine measured in the 12 mo before hospitalization if available; otherwise, this was the lowest plasma creatinine measured between hospitalization and ICU admission. An estimated creatinine level was used for patients for whom there was no level available from the 12 mo before hospitalization to the time of ICU admission. The baseline creatinine was estimated for 14 patients (1.4%) in the lactated Ringers group and 17 patients (1.6%) in the Normosol-R group.

**Figure 2:**
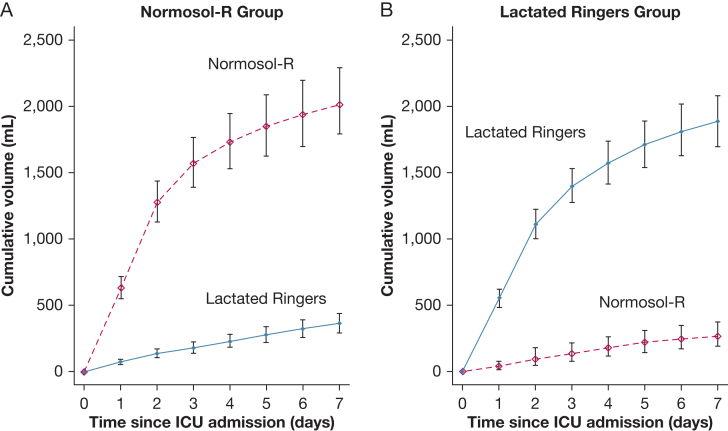
A, B, Volume of IV isotonic balanced crystalloid administered according to group. The mean cumulative volume of Normosol-R (dotted line) and lactated Ringers (solid line) between admission to the ICU and hospital discharge is shown for patients in the Normosol-R (A) and lactated Ringers group (B). I bars indicate 95% CIs.

Among patients admitted through the emergency department, between presentation to the emergency department and admission to the ICU, a total of 134 patients (21.8%) in the Normosol-R group received Normosol-R, compared with 25 patients (3.9%) in the lactated Ringers group; the mean volume ± SD was 301 ± 737 mL in the Normosol-R group and 63 ± 518 mL in the lactated Ringers group. A total of 311 patients (50.6%) in the Normosol-R group received lactated Ringers, compared with 378 patients (58.6%) in the lactated Ringers group; the mean volume ± SD was 784 ± 1,127 mL in the Normosol-R group and 954 ± 1,199 mL in the lactated Ringers group. Between ICU admission and the first of hospital discharge or 30 days, the volume of Normosol-R administered to patients in the Normosol-R group was a median of 800 mL (IQR, 0-2,600) and a mean ± SD of 2,333 ± 5,214 mL. The volume of lactated Ringers solution administered to patients in the lactated Ringers group was a median of 875 mL (IQR, 0-3,000) and a mean ± SD of 2,327 ± 4,689 mL (e-Tables 2, 3; Fig 2). Between ICU admission and the first of hospital discharge or 30 days, the median volume of 0.9% sodium chloride received on each study day in each group was 0 mL (IQR, 0-0) (e-Table 4).

**Figure 3:**
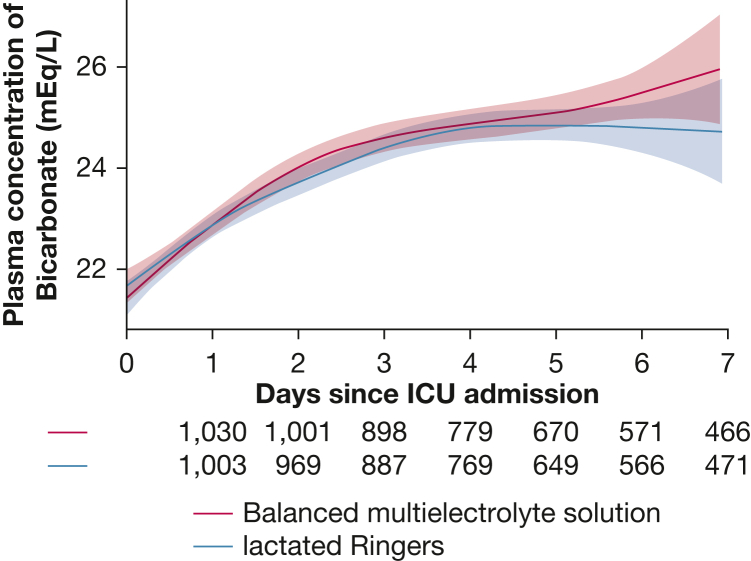
Plasma HCO_3_^−^ concentration according to group. The mean and 95% CI (denoted by gray shading) for the plasma HCO_3_^−^ concentration on the first 7 d since admission to the ICU are shown for patients in the Normosol-R group and lactated Ringers group with locally weighted scatterplot smoothing. HCO_3_^−^ = bicarbonate.

In the primary analysis adjusting for prespecified covariates, the plasma HCO_3_^−^ concentration between enrollment and day 7 did not differ between patients in the Normosol-R group and the lactated Ringers group (mean difference, −0.12 mmol/dL; 95% CI, −0.61 to 0.36; *P* = .61) (Fig 3). Results were similar in an unadjusted analysis (mean difference, −0.11; 95% CI, −0.60 to 0.37) (e-Table 5) and in prespecified sensitivity analyses (e-Table 6). The primary outcome did not differ significantly between the Normosol-R group and the lactated Ringers group in any of the prespecified subgroups, including among patients who received larger volumes of IV crystalloid (e-Figs 1-3, e-Table 7). The percentage of patients who experienced a plasma HCO_3_^−^ concentration < 20 mM between enrollment and 7 days did not differ between the Normosol-R group (50.8%) and the lactated Ringers group (48.4%) (absolute risk difference, 2.4%; 95% CI, −2.0% to 6.9%). The lowest plasma HCO_3_^−^ concentration was also similar between the Normosol-R group (median, 19 mM; IQR, 16-23) and the lactated Ringers group (median, 20 mM; IQR, 16-23) (Table 2).

**Table 2:** Secondary Outcomes

Outcome	No.	Normosol-R	Lactated Ringers	Absolute or Median Difference (95% CI)
		(n = 1,056)	(n = 1,028)	
Secondary laboratory outcomes^a^				
Lowest HCO_3_^−^concentration, mM	2,033	19 (16 to 23)	20 (16 to 23)	−1 (−3 to −1)
HCO_3_^−^ concentration < 20 mM	2,033	523 (50.8)	485 (48.4)	2.4 (−2.0 to 6.9)
Highest chloride concentration, mM	2,031	108 (104 to 111)	108 (104 to 111)	0 (−1 to 1)
Change in chloride concentration from enrollment to peak, mM	2,010	3 (1 to 7)	3 (1 to 7)	0 (−1 to 1)
Chloride concentration < 100 mM	2,031	390 (37.8)	387 (38.7)	−0.9 (−5.2 to 3.5)
Chloride concentration > 110 mM	2,031	312 (30.3)	309 (30.9)	−0.6 (−4.7 to 3.5)
Sodium concentration > 145 mM	2,031	133 (12.9)	132 (13.2)	−0.3 (−3.3 to 2.7)
Sodium concentration < 135 mM	2,031	463 (44.9)	439 (43.9)	1.0 (−3.4 to 5.4)
Potassium concentration > 5.5 mM	2,033	219 (21.2)	198 (19.8)	1.5 (−2.1 to 5.1)
Mean strong ion difference, mM^b^^,^^c^	490	42.1 (38.2 to 45.5)	41.8 (38.3 to 45.1)	0.4 (−0.9 to 1.3)
Mean arterial pH^c^	539	7.4 (7.3 to 7.4)	7.4 (7.3 to 7.4)	−0.0 (−0.0 to 0.0)
Mean base excess^c^	532	−1.4 (−6.0 to 2.5)	−1.4 (−6.0 to 2.8)	−0.0 (−1.5 to 1.1)
Additional kidney outcomes				
Major adverse kidney event within 30 d^d^	2,084	289 (27.4)	260 (25.3)	2.1 (−1.8 to 6.0)
Receipt of new kidney replacement therapy^e^	1,916	58 (6.0)	47 (5.0)	1.0 (−1.2 to 3.1)
Stage II or higher acute kidney injury^e^	1,916	330 (33.9)	288 (30.6)	3.3 (−1.0 to 7.6)
Final creatinine ≥ 200% of baseline^e^	1,839	40 (4.3)	29 (3.2)	1.1 (−0.8 to 2.9)
Creatinine, mg/dL^e^				
Highest before discharge or 30 d	1,862	1.2 (0.8 to 2.4)	1.3 (0.9 to 2.4)	−0.0 (−0.1 to 0.1)
Change from baseline to highest value	1,839	0.1 (−0.1 to 0.3)	0.1 (−0.1 to 0.3)	0.0 (−0.0 to 0.0)
Final value before discharge or 30 d	1,862	0.9 (0.7 to 1.5)	0.9 (0.7 to 1.4)	0.0 (−0.0 to 0.1)
Secondary clinical outcomes				
30-d in-hospital mortality	2,084	172 (16.3)	164 (16.0)	0.3 (−2.9 to 3.6)
ICU-free days^f^	2,084	24.0 (19.0 to 26.0)	24.0 (20.0 to 26.0)	0.0 (0.0 to 1.0)
Ventilator-free days^f^	2,084	28.0 (22.0 to 28.0)	28.0 (23.0 to 28.0)	0.0 (0.0 to 0.0)
Vasopressor-free days^f^	2,084	28.0 (25.0 to 28.0)	28.0 (26.0 to 28.0)	0.0 (0.0 to 0.0)
Kidney-replacement therapy-free days^f^	2,084	28.0 (26.0 to 28.0)	28.0 (28.0 to 28.0)	0.0 (0.0 to 0.0)

Continuous data are presented as median (25th percentile-75th percentile) or mean (SD); other values are No. (%) or as otherwise indicated. HCO_3_^−^ = bicarbonate.

a Between enrollment and 7 d.

b Strong ion difference calculated by [(sodium + potassium + calcium) – (chloride + lactate)].

c Values reported are the median of each patient’s mean value (strong ion difference, arterial pH, base excess).

d Major adverse kidney events within 30 d are the composite of death, receipt of new kidney replacement therapy, or final creatinine ≥ 200% baseline, all censored at the first of hospital discharge or 30 d after ICU admission.

e Receipt of new kidney replacement therapy and outcomes based on creatinine measurements are only reported among the 1,916 patients (974 patients in the Noromosol-R group and 942 in the lactated Ringers group) who had not received kidney replacement therapy before ICU admission.

f ICU-, ventilator-, vasopressor-, and kidney replacement therapy-free days refer to the number of days alive and free from the specified therapy in the first 28 d after ICU admission.

The Normosol-R and lactated Ringers groups had a similar percentage of patients with plasma chloride concentrations > 110 mM from enrollment to day 7 (30.3% vs 30.9%; absolute risk difference, −0.6%, 95% CI, −4.7% to 3.5%), percentage of patients with plasma chloride concentrations < 100 mM (37.8% vs 38.7%; absolute risk difference, −0.9%; 95% CI, −5.2% to 3.5%), and change in plasma chloride concentration from enrollment to the highest value (median, 3; IQR, 1-7 in both trial groups).

The Normosol-R and lactated Ringers groups were similar regarding the percentage of patients with a plasma sodium concentration > 145 mM (12.9% vs 13.2%; absolute risk difference, −0.3%; 95% CI, −3.3% to 2.7%) (e-Fig 4), with a plasma sodium concentration < 135 mM (44.9% vs 43.9%; absolute risk difference, 1.0%; 95% CI, −3.4% to 5.4%), and with a plasma potassium concentration > 5.5 mM (21.2% vs 19.8%; absolute risk difference, 1.5%; 95% CI, −2.1% to 5.1%). The median plasma strong ion difference was 42.1 mM (IQR, 38.2-45.5) in the Normosol-R group and 41.8 mM (IQR, 38.3-45.1) in the lactated Ringers group (median difference, 0.4; 95% CI, −0.9 to 1.3) (e-Fig 5).

A total of 289 patients (27.4%) in the Normosol-R group and 260 patients (25.3%) in the lactated Ringers group experienced a major adverse kidney event by day 30 (absolute risk difference, 2.1%; 95% CI, −1.8% to 6.0%). In-hospital mortality by day 30 occurred in 172 patients (16.3%) in the Normosol-R group and 164 patients (16.0%) in the lactated Ringers group (absolute risk difference, 0.3%; 95% CI, −2.9% to 3.6%) (e-Fig 6). A total of 58 patients (6.0%) in the Normosol-R group and 47 patients (5.0%) in the lactated Ringers group received new kidney replacement therapy by day 30 (absolute risk difference, 1.0%; 95% CI, −1.2% to 3.1%). The number of vasopressor-free, ventilator-free, and ICU-free days in each group is shown in Table 2.

## Discussion

Among critically ill adults in this randomized trial, the choice of balanced crystalloid solution for IV fluid therapy did not result in differences in plasma HCO_3_^−^ concentration, organ function, or clinical outcomes. These findings have implications for clinical care because (1) previous trials suggest that using balanced crystalloids rather than saline may improve outcomes for critically ill adults, (2) balanced crystalloid solutions like Normosol-R and lactated Ringers represent the 2 types of balanced crystalloid widely available and administered in current clinical care, and (3) to our knowledge, no previous large trials have compared solutions like Normosol-R and solutions like lactated Ringers among critically ill adults.

Previous randomized trials examining the effect of balanced crystalloid composition have ranged in size from 14 to 204 participants and have primarily examined fluid therapy in the operating room during surgery.^15^ Together, these studies suggest that use of solutions like Normosol-R resulted in lower plasma chloride concentrations and higher base excess compared with lactated Ringers solution. The effect of balanced crystalloid composition on pH and HCO_3_^−^ concentration were inconsistent between trials. Among the 2,084 critically ill adults in our trial, plasma concentrations of sodium, chloride, and HCO_3_^−^ were similar between the Normosol-R and lactated Ringers groups. The results of our trial may have differed from previous trials in the operating room through differences in the patient populations (eg, electrolyte derangements of critical illness vs elective surgery), in the time periods over which fluid therapy was infused, or in the co-interventions received (eg, receipt of IV fluid from medication therapy in the ICU).

Our trial did not detect the differences in HCO_3_^−^ concentration or acid-base balance that we hypothesized would occur based on the total amount of buffer contained in Normosol-R (50 mM) compared with lactated Ringers (28 mM). Several potential explanations for this exist. First, the gluconate in Normosol-R may be excreted unchanged in the urine rather than being metabolized to an effective buffer.^16^ If so, then the concentration of effective buffers in Normosol-R (27 mM acetate) and lactated Ringers (28 mM lactate) are sufficiently similar that the effect on patients’ plasma acid-base balance would not be expected to differ between the solutions. Second, the differing pathways by which acetate and lactate undergo metabolism may affect to what degree they alkalinize patients’ plasma.^17^ Third, the hyperchloremic acidosis after saline infusion has been attributed to the high concentration of chloride in saline (154 mM) compared with human plasma (98-108 mM). Because the difference in chloride concentration between Normosol-R (98 mM) and lactated Ringers (109 mM) is much smaller than the difference between balanced crystalloids and saline, any effect of chloride administration on acid-base balance might differ less between the 2 balanced crystalloid solutions than between balanced crystalloid solutions and saline.

Our trial has several strengths. Group assignment at the level of the ICU allowed delivery of the assigned IV fluid solution early in each patient’s critical illness. Enrolling all adults admitted to the participating ICU and allowing clinical providers to deliver the assigned crystalloid during clinical care minimized selection bias and improved generalizability. Compliance with the assigned balanced crystalloid was high.

The trial also has limitations. Conduct in a single ICU at a single academic center limits generalizability. Treating clinicians were aware of the composition of the assigned balanced crystalloid and of the group-assignment sequence. Although the primary outcome of HCO_3_^−^ level is objective, a clinician could adjust their treatment in response, which may introduce the possibility of treatment bias. Additionally, although a change in serum HCO_3_^−^ is not a patient-centered outcome, the absence of even small differences between fluids in serum electrolyte concentrations make differences in downstream patient-centered outcomes unlikely, and patient-centered secondary outcomes in our trial were numerically similar between groups. Furthermore, this trial’s sample size of 2,084 patients may not have been sufficient to detect small differences between trial groups in serum HCO_3_^−^ concentration. In the current trial, the choice of crystalloid solution was controlled by the trial beginning when patients arrived in the study ICU or when admission to the study ICU was planned for a patient located in the hospital’s emergency department. Thus, fluid therapy was not consistently controlled for patients very early in critical illness, which prior studies have suggested may be a critical period for the effect of fluid composition on outcomes. Finally, the total volume of study fluid administered between ICU admission and hospital discharge was low; however, similar volumes were sufficient to influence plasma HCO_3_^−^ and electrolyte concentrations in prior trials of balanced crystalloid compared with saline and these volumes are reflective of the volumes administered in current clinical care.^7^

## Interpretation

In conclusion, among critically ill adults in this trial, use of Normosol-R rather than lactated Ringers for IV fluid therapy did not affect patients’ plasma HCO_3_^−^ concentrations or clinical outcomes.

## Funding/Support

This project was supported by the VICTR Learning Healthcare System Platform [CTSA Award UL1 TR002243] from the National Center for Advancing Translational Sciences. E. T. Q. was supported by the 10.13039/100000050National Heart, Lung, and Blood Institute (NHLBI) [Grant T32HL087738]. M. W. S. was supported in part by the NHLBI [Grant K23HL143053]. R. E. F. was supported by the NHLBI [Grant K23HL148640].

## Financial/Nonfinancial Disclosures

The authors have reported to *CHEST* the following: E. T. Q. reports financial support provided by the National Institutes of Health. T. W. R. reports financial support provided by the National Institutes of Health and serves in an editorial capacity for *CHEST*. R. E. F. reports financial support provided by the National Center for Advancing Translational Sciences and the National Institutes of Health, reports relationships with Oak Hill Clinical Informatics and Phillips Healthcare that includes consulting or advisory, reports a relationship with Hall Booth Smith that includes paid expert testimony, and reports a relationship with the Society for Technology in Anesthesia that includes board membership. J. D. C. reports relationships with the National Institutes of Health, the US Department of Defense, and the Patient-Centered Outcomes Research Institute that includes funding grants; and a relationship with Fisher & Paykel Healthcare Inc that includes travel reimbursement. M. W. S. reports relationships with the National Institutes of Health, the US Department of Defense, and the Patient-Centered Outcomes Research Institute that includes funding grants; and reports a relationship with Baxter Healthcare that includes consulting or advisory and speaking and lecture fees. E. D. S. reports a relationship with the National Institute of Diabetes and Digestive and Kidney Diseases that includes funding grants, reports a relationship with UptoDate Inc that includes consulting or advisory, and reports a relationship with the *Clinical Journal of the American Society of Nephrology* that includes board membership. None declared (R. M. B., K. E. J., L. W., J. L. S., J. P. W., G. R. B., W. H. S.).

## Supplementary Data

e-Online Data
