# Supplementary material for: Normosol-R vs Lactated Ringers in the Critically Ill: A Randomized Trial
Source: Chest. 2025 Feb 17;168(2):336–45. doi: 10.1016/j.chest.2025.02.008 (PMC12405922; doi:10.1016/j.chest.2025.02.008)
Supplement: e-Online Data [file mmc1.docx]

Balanced Multielectrolyte Solution versus Lactated Ringers in Critically Ill Adults: A Randomized Trial

Supplementary Appendixes

**Contents**

[**SUPPLEMENTAL METHODS** 3](#_Toc187760626)

[IRB Approval and Waiver of Informed Consent 3](#_Toc187760627)

[Inclusion and Exclusion Criteria 4](#_Toc187760628)

[Composition of study fluids 6](#_Toc187760629)

[Block Randomization Schedule for the participating ICU’s assignment to Normosol-R versus Lactated Ringer’s. 7](#_Toc187760630)

[Trial Outcomes 8](#_Toc187760631)

[Assessment of Baseline Creatinine 9](#_Toc187760632)

[Assessment of Free Days Outcomes 10](#_Toc187760633)

[Sample Size and Sample Size Re-estimation 11](#_Toc187760634)

[Sensitivity Analyses 12](#_Toc187760635)

[Effect Modification 13](#_Toc187760636)

[Handling of Missing Data 14](#_Toc187760637)

[**SUPPLEMENTAL FIGURES** 15](#_Toc187760638)

[Figure S1. Effect Modification of the Primary Outcome 15](#_Toc187760639)

[Figure S2. Effect Modification of the Primary Outcome by Baseline Bicarbonate 16](#_Toc187760640)

[Figure S3. Effect Modification of the Primary Outcome by Volume Received 17](#_Toc187760641)

[Figure S4. Effect Modification of Serum Sodium by Volume Received 18](#_Toc187760642)

[Figure S5. Correlation between strong ion difference and bicarbonate concentration 19](#_Toc187760643)

[Figure S6. Effect Modification of Mortality 20](#_Toc187760644)

[**SUPPLEMENTAL TABLES** 21](#_Toc187760645)

[Table S1. First Daily Lab Values 21](#_Toc187760646)

[Table S2. Median Daily Cumulative Crystalloid Volumes 24](#_Toc187760647)

[Table S3. Mean Daily Cumulative Crystalloid Volumes 25](#_Toc187760648)

[Table S4. Daily 0.9% Sodium Chloride Volumes 26](#_Toc187760649)

[Table S5. Multivariate Modeling of the Primary Outcome 27](#_Toc187760650)

[Table S6. Sensitivity Analyses of the Primary Outcome 28](#_Toc187760651)

[Table S7. Effect Modification of the Primary Outcome 29](#_Toc187760652)

[**SUPPLEMENTAL REFERENCES** 30](#_Toc187760653)

### **SUPPLEMENTAL METHODS**

## **IRB Approval and Waiver of Informed Consent**

Normosol-R solution and lactated Ringer’s solution are intravenous balanced crystalloid solutions currently used in the routine care of patients admitted to the medical ICU at Vanderbilt University Medical Center. Recent data have demonstrated benefit with the use of balanced crystalloids compared to saline, however, no high-quality data suggest that choice among the two available types of balanced crystalloids affects clinical outcomes among critically ill adults. During this trial, each time a isotonic crystalloid was ordered, the study confirmed that the treating clinician (1) did not feel that an isotonic crystalloid not included in the study (ex: 0.9% sodium chloride) was required for the safe treatment of that specific patient at that specific point in time and (2) did not feel the non-assigned balanced crystalloid was required for the safe treatment of that specific patient at that specific point in time.

The trial was felt to pose minimal risk because (1) exposure to the study crystalloids occurred only for patients whose treating clinician had already decided to administer an intravenous (IV) isotonic crystalloid, (2) all of the crystalloid solutions examined were already used in routine practice in the study environment, (3) the fluids had been proven to improve clinical outcomes compared to saline (the historically used fluid in the study environment), (4) no definitive prior data suggested clinical outcomes were better with one balanced crystalloid relative to the other, and (5) treating clinicians were allowed to use a non-assigned crystalloid during the trial if felt to be required for the optimal treatment of a specific patient. Given the minimal risk, the focus of the study on crystalloid use at an ICU level, and the impracticability of consenting each patient admitted to the ICU prior to the first administration of crystalloid, a waiver of informed consent was requested from and approved by the institutional review board at Vanderbilt University Medical Center (reference number 180397).

## **Inclusion and Exclusion Criteria**

The inclusion criteria for the study were:

1. Located in the medical intensive care unit during the study period

The exclusion criteria for the study were:

1. Age < 18 years old

Consistent with regulations from the Institutional Review Board overseeing the trial, data from any patients who were determined to be prisoners were immediately removed from the trial dataset. No data from prisoners were included in the trial dataset or analyses.

## **Composition of study fluids**

| Concentration (mmol/L) | | | | |
| --- | --- | --- | --- | --- |
|  | Plasma | 0.9% NaCl | Lactated Ringer’s | Normosol-R® |
| Sodium | 140 | 154 | 130 | 140 |
| Potassium | 5 | 0 | 4 | 5 |
| Chloride | 100 | 154 | 109 | 98 |
| Calcium | 2.2 | 0 | 1.5 | 0 |
| Magnesium | 1 | 0 | 0 | 1.5 |
| Bicarbonate | 24 | 0 | 0 | 0 |
| Lactate | 1 | 0 | 28 | 0 |
| Acetate | 0 | 0 | 0 | 27 |
| Gluconate | 0 | 0 | 0 | 23 |
| Strong ion difference | 42 | 0 | 28 | 50 |

## **Block Randomization Schedule for the participating ICU’s assignment to Normosol-R versus Lactated Ringer’s.**


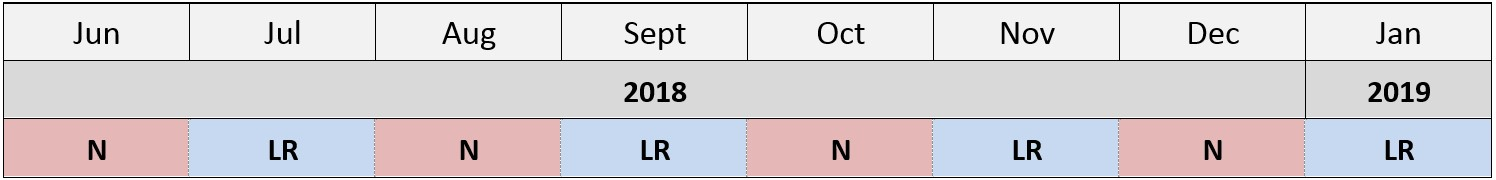


For each of the 8 month-long blocks, the participating intensive care unit (ICU) was assigned to use either Normosol-R solution (N) or lactated Ringer’s solution (LR). Simple randomization was used to generate the group assignment for the initial block. Trial group assignment then alternated monthly between the two study fluids for the remaining blocks.

## **Trial Outcomes**

**Primary Outcome**

The primary outcome was the plasma bicarbonate concentration over the first 7 days after ICU admission.

**Secondary Outcomes**

Secondary laboratory outcomes:

- From enrollment to 7 days after enrollment:
  - Plasma bicarbonate concentration less than 20 mmol/L
  - Lowest bicarbonate concentration (mmol/L)
  - Chloride concentration > 110 mmol/L
  - Chloride concentration < 100 mmol/L
  - Highest chloride concentration (mmol/L)
  - Change in chloride concentration from enrollment to peak (mmol/L)
  - Sodium concentration > 145 mmol/L
  - Sodium concentration < 135 mmol/L
  - Potassium concentration > 5.5 mmol/L
  - Lowest arterial pH
  - Standard Base Excess
- Daily sodium, potassium, chloride, bicarbonate, blood urea nitrogen, creatinine, calcium, and lactate values from enrollment to 30 days after enrollment or hospital discharge
- Correlation between measured strong ion difference and bicarbonate value.

Secondary clinical outcomes

- Major Adverse Kidney Events by day 30 (MAKE30) defined as the composite of:
  - In-hospital mortality by day 30
  - Receipt of new kidney replacement therapy by day 30
  - Persistent renal dysfunction by day 30 (final serum creatinine before day 30 or discharge ≥ 200% of baseline)
- Stage II or higher acute kidney injury by Kidney Disease: Improving Global Outcomes creatinine criteria^1^
- Intensive care unit-free days through 28 days after enrollment
- Ventilator free-days through 28 days after enrollment
- Vasopressor-free days through 28 days after enrollment
- Kidney replacement therapy -free days through 28 days after enrollment

## **Assessment of Baseline Creatinine**

The value for baseline serum creatinine was determined using a previously described hierarchical approach^2^. The lowest serum creatinine between 12 months and 24 h prior to hospital admission was used when available. If no such creatinine value was available, the lowest creatinine value between 24 h prior to hospital admission and the time of ICU admission was used. If no creatinine value was available between 12 months prior to hospital admission and the time of ICU admission, a baseline creatinine value was estimated using a previously-described formula [creatinine = 0.74 − 0.2 (if female) + 0.08 (if African American) + 0.003 × age (in years)]^3^. Patients known to have received kidney replacement therapy prior to enrollment were considered ineligible to meet criteria for new kidney replacement therapy or persistent renal dysfunction, but could qualify for MAKE30 by experiencing in-hospital mortality.

## **Assessment of Free Days Outcomes**

The number of days alive and free of a supportive therapy (e.g., kidney replacement therapy-free days, ventilator-free days, and vasopressor-free days) was defined as the number of calendar days alive and free of the supportive therapy from the final receipt of the supportive therapy through day 28 after enrollment. The day of enrollment was considered to be day 0. Outcome ascertainment ceased at the time of hospital discharge or the specified day after enrollment, whichever occurred first. Receipt of the supportive therapy was considered to end at the time of the patient’s final receipt of the supportive therapy between enrollment and the specified day after enrollment. Patients who continued to receive the supportive therapy at the specified day after enrollment received a value of zero. Patients who died prior to or on the specified day after enrollment received a value of zero. Patients discharged from the hospital prior to the specified day after enrollment who were receiving the supportive therapy at the time of discharge received a value of zero. Patients who were removed from the supportive therapy and were discharged from the hospital without the supportive therapy prior to the specified day after enrollment were assumed to remain free of the supportive therapy between hospital discharge and the specified day after enrollment. For patients removed from the supportive therapy, returned to receiving the supportive therapy, and subsequently removed again prior to the specified day after enrollment, days alive and free of the supportive therapy were counted from the final receipt of the supportive therapy prior to the specified day after enrollment.

ICU-free days through study day 28: Number of days alive and free of the ICU were defined as the number of calendar days in which the patient was alive between the patient’s final transfer or discharge from an ICU service and day 28 after enrollment. Patients who were never discharged from the intensive care unit received a value of 0. Patients who died before or on day 28 received a value of 0. Patients who returned to an intensive care unit service and were subsequently discharged prior to day 28, ICU-free days were considered to begin the day following the date of final ICU discharge. All data were censored at hospital discharge or 28 days, whichever came first.

Hospital-free days to study day 28: Number of days alive and free of the hospital were defined as the number of calendar days between enrollment and 28 days after enrollment, in which the patient was alive and not admitted to the hospital. Patients who were never discharged from the hospital received a value of 0. Patients who died before day 28 received a value of 0. All data were censored at hospital discharge from the hospitalization in which they were enrolled in the trial or 28 days, whichever came first.

## **Sample Size and Sample Size Re-estimation**

This trial aimed to evaluate the effect of balanced crystalloid composition on serum bicarbonate concentration. A prior cluster-crossover pilot study in the same study ICU comparing saline to balanced crystalloids enrolled 974 patients over a four-month study duration^2^. As a cluster-crossover trial, the study duration was set and the actual enrollment depended on the observed number of qualifying admissions to the study ICU during the study period. We anticipated this would be greater than 1,800 patients and no more than 2,400 patients.

The mean bicarbonate concentration within 7 days of ICU admission among in the patients admitted to the medical ICU in the aforementioned trial was 24 +/- 6 mmol/L. We calculated that enrolling 1,800 patients would provide 90% statistical power with a two-sided alpha of 0.05 to detect a difference of 0.9 mmol/L in mean bicarbonate level assuming common standard deviation of 6 mmol/L.

## **Sensitivity Analyses**

To assess robustness of findings, the main analysis of the primary outcome was repeated in two alternative populations.

1. Excluding week before crossover (“washout period”). The primary analysis was repeated excluding those admitted in the 7 days prior to a crossover in ICU crystalloid assignment (simulating a “washout” period). Prior data from the study ICUs suggests that less than 10% of patients remain in the ICU for longer than 7 days. Excluding those admitted within 7 days of a crossover in ICU crystalloid assignment will allow use of a baseline factor to exclude the majority of patients who would go on to experience a crossover in crystalloid assignment due to the study design.
2. First medical ICU admission only. The primary analysis was repeated including only the first medical ICU admission in the study for each patient.

## **Effect Modification**

Pre-specified baseline variables were evaluated for whether they modified the effect of study group assignment (Normosol-R versus lactated Ringer’s) on the primary outcome and the outcome of in-hospital mortality using a formal test of statistical interaction in an unadjusted model with the outcome as the dependent variable and independent variables for study group, the pre-specified proposed effect modifier, and the interaction between the two. For categorical variables, the odds ratio and 95% confidence intervals are presented within each pre-specified subgroup. In accordance with the Instrument for assessing the Credibility of Effect Modification Analyses (ICEMAN) recommendations, the following limited number of baseline variables were prespecified as potential effect modifiers:

1. Baseline bicarbonate concentration (continuous variable)
2. Source of admission to the ICU (Emergency department, Transfer from another hospital, Hospital ward, Other ICU, Operating room, Other)
3. Sepsis or septic shock (Yes, No)
4. Cirrhosis (Yes, No)
5. Receipt of mechanical ventilation (Yes, No)
6. Receipt of vasopressors (Yes, No)
7. Category of renal dysfunction at the time of enrollment (No renal dysfunction, Acute kidney injury, Chronic kidney disease, End-stage renal disease receiving RRT)
8. Severity of Illness (SOFA score greater than 5, 5 or lower) – the originally specified severity of illness score (UHC expected mortality) ceased to be available during the study period and was replaced with the SOFA score^4^.
9. Volume of isotonic crystalloid received

## **Handling of Missing Data**

No data were missing regarding death or the receipt of kidney replacement therapy. Patients could be missing data for bicarbonate values between enrollment and the first of hospital discharge or 30 days. When data were missing for primary, secondary, or exploratory outcomes, complete-case analysis was performed; no data on outcomes were imputed. In adjusted analyses, missing data for baseline covariates were imputed using multiple imputations.

### **SUPPLEMENTAL FIGURES**

## **Figure S1. Effect Modification of the Primary Outcome**


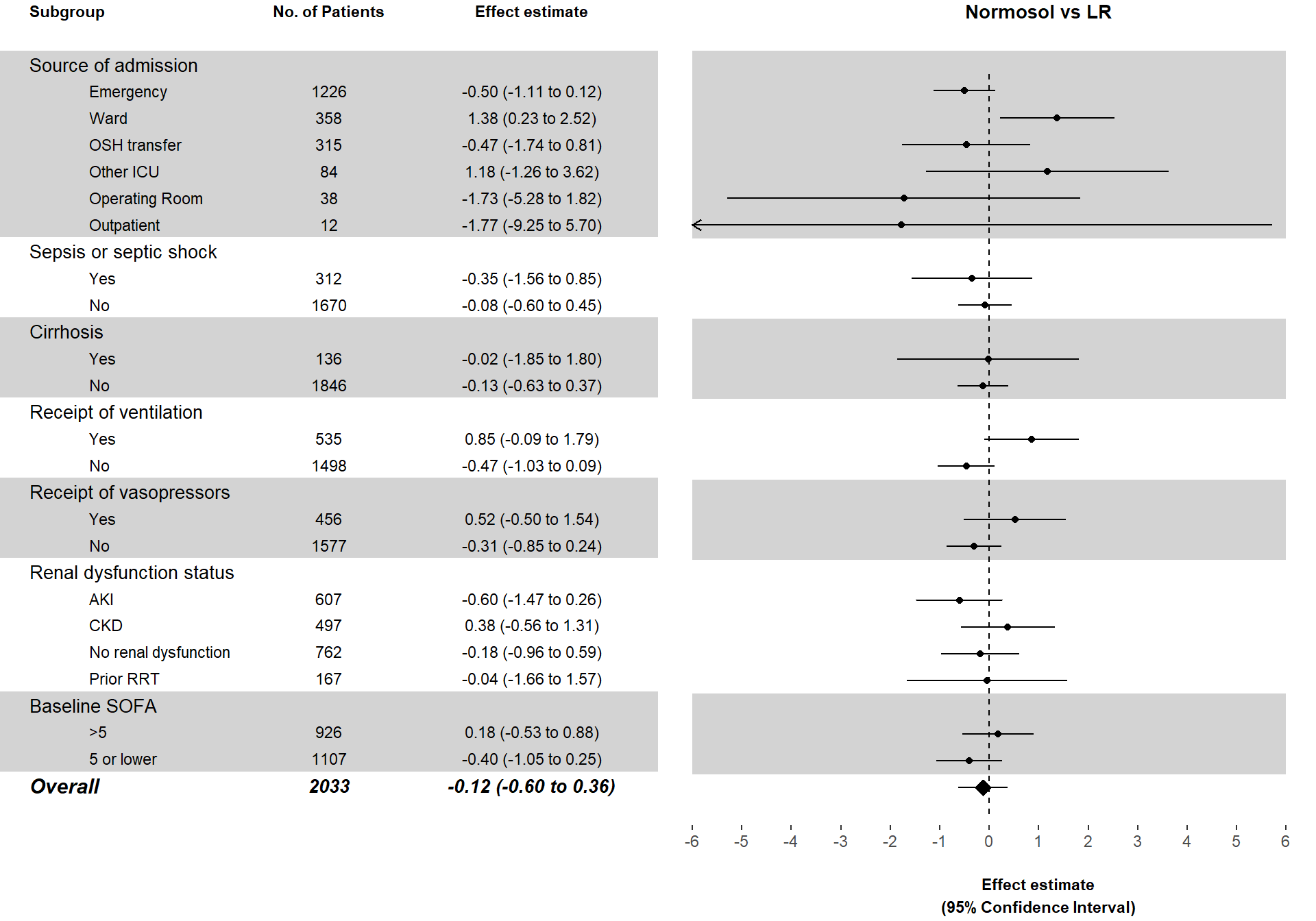


This figure displays the difference in bicarbonate concentration (mmol/dL) between the lactated Ringer’s group (LR) and the Normosol-R. A value of 0 mmol/dL indicates no difference between groups, values less than 0 indicate a higher bicarbonate concentration with lactated Ringer’s and values greater than 0 indicate a higher bicarbonate concentration with Normosol. For sources of admission, Emergency refers to Emergency Department, Ward refers to a non-ICU floor within the hospital, and OSH transfer refers to a transfer from an outside hospital. AKI is acute kidney injury; CKD is chronic kidney disease, and RRT is renal replacement therapy. SOFA is the sequential organ failure assessment score.

## **Figure S2. Effect Modification of the Primary Outcome by Baseline Bicarbonate**


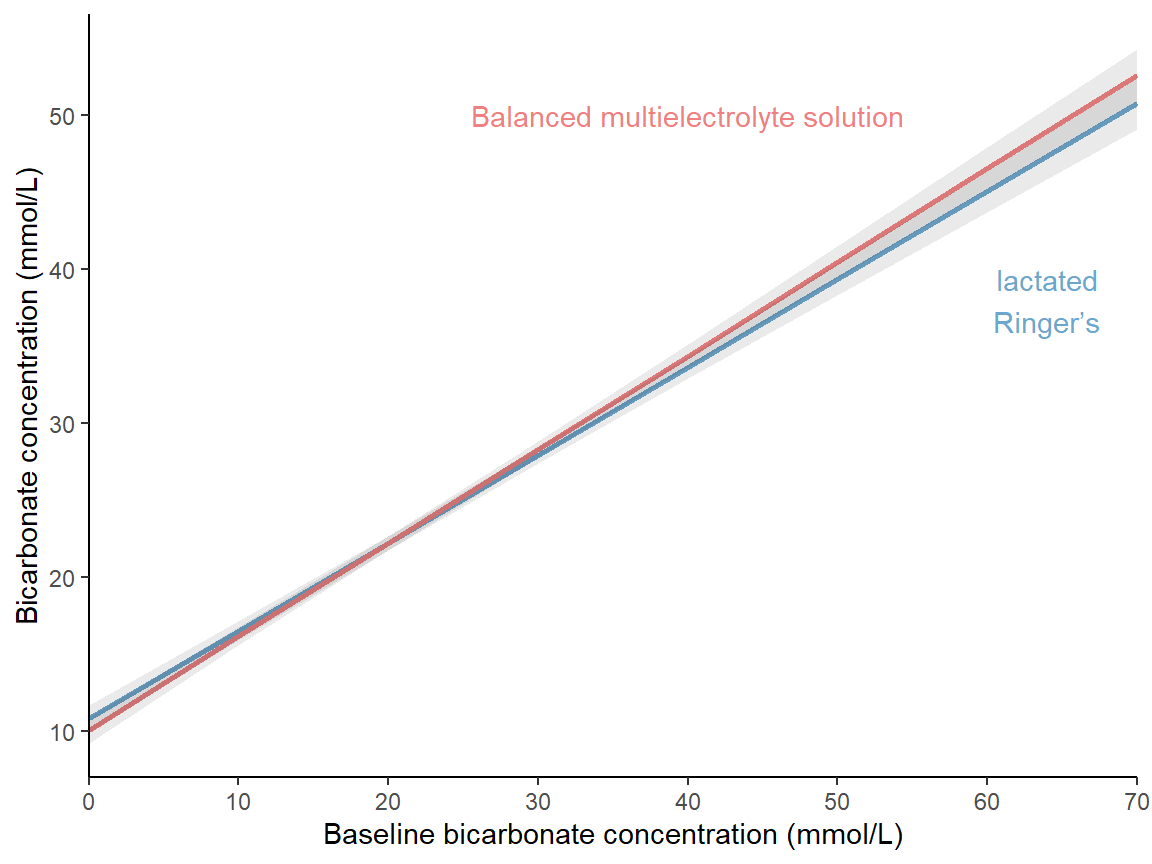


This figure displays the day 7 bicarbonate concentration (mmol/dL) between the lactated Ringer’s group (LR) and the Normosol-R on the y-axis compared to the baseline bicarbonate concentration on the x-axis.

## **Figure S3. Effect Modification of the Primary Outcome by Volume Received**


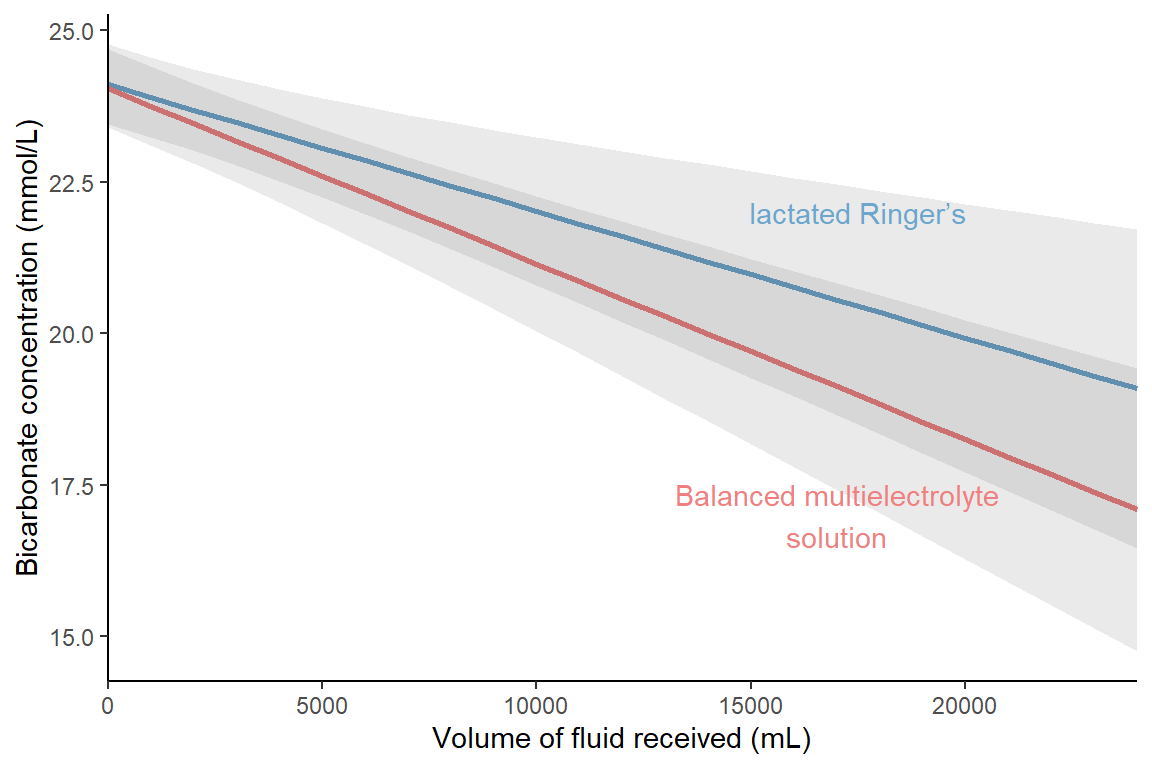


This figure displays the day 7 bicarbonate concentration (mmol/dL) between the lactated Ringer’s group (LR) and the Normosol-R on the y-axis compared to cumulative volume on the x-axis.

## **Figure S4. Effect Modification of Serum Sodium by Volume Received**


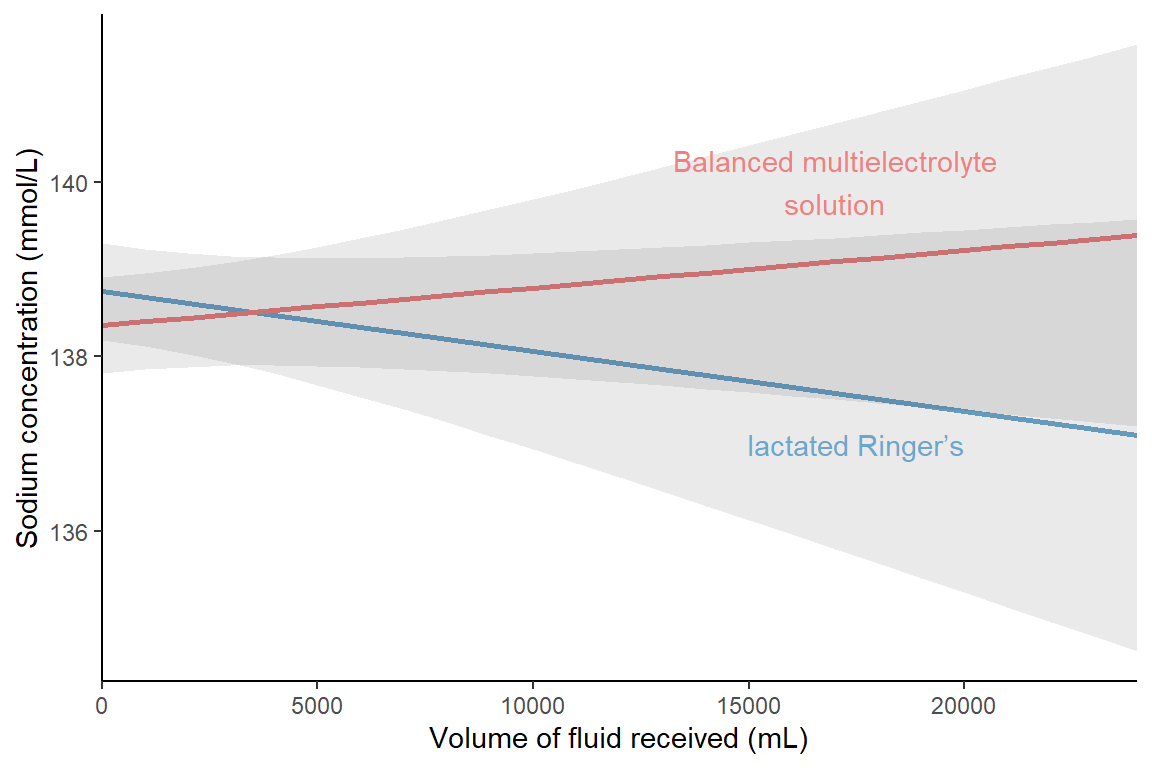


This figure displays the day 7 bicarbonate concentration (mmol/dL) between the lactated Ringer’s group (LR) and the Normosol-R on the y-axis compared to cumulative volume on the x-axis.

## **Figure S5. Correlation between strong ion difference and bicarbonate concentration**


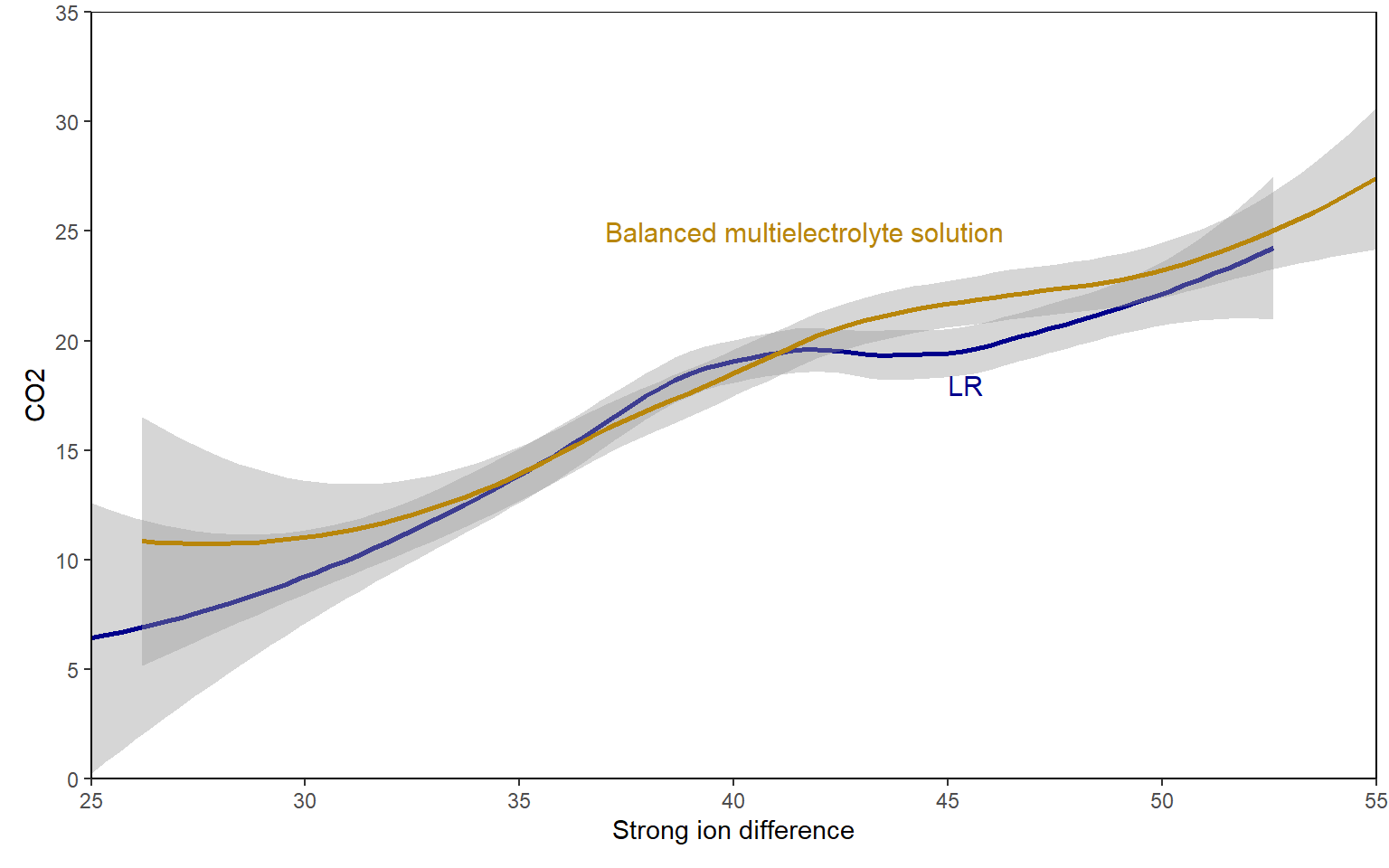


This figure displays the association between the strong ion difference (calculated as [sodium + potassium + calcium] – [chloride + lactate]) on the X axis and the plasma bicarbonate concentration (CO2) on the Y axis in the Normosol-R group and the lactated Ringer’s (LR) group.

## **Figure S6. Effect Modification of Mortality**


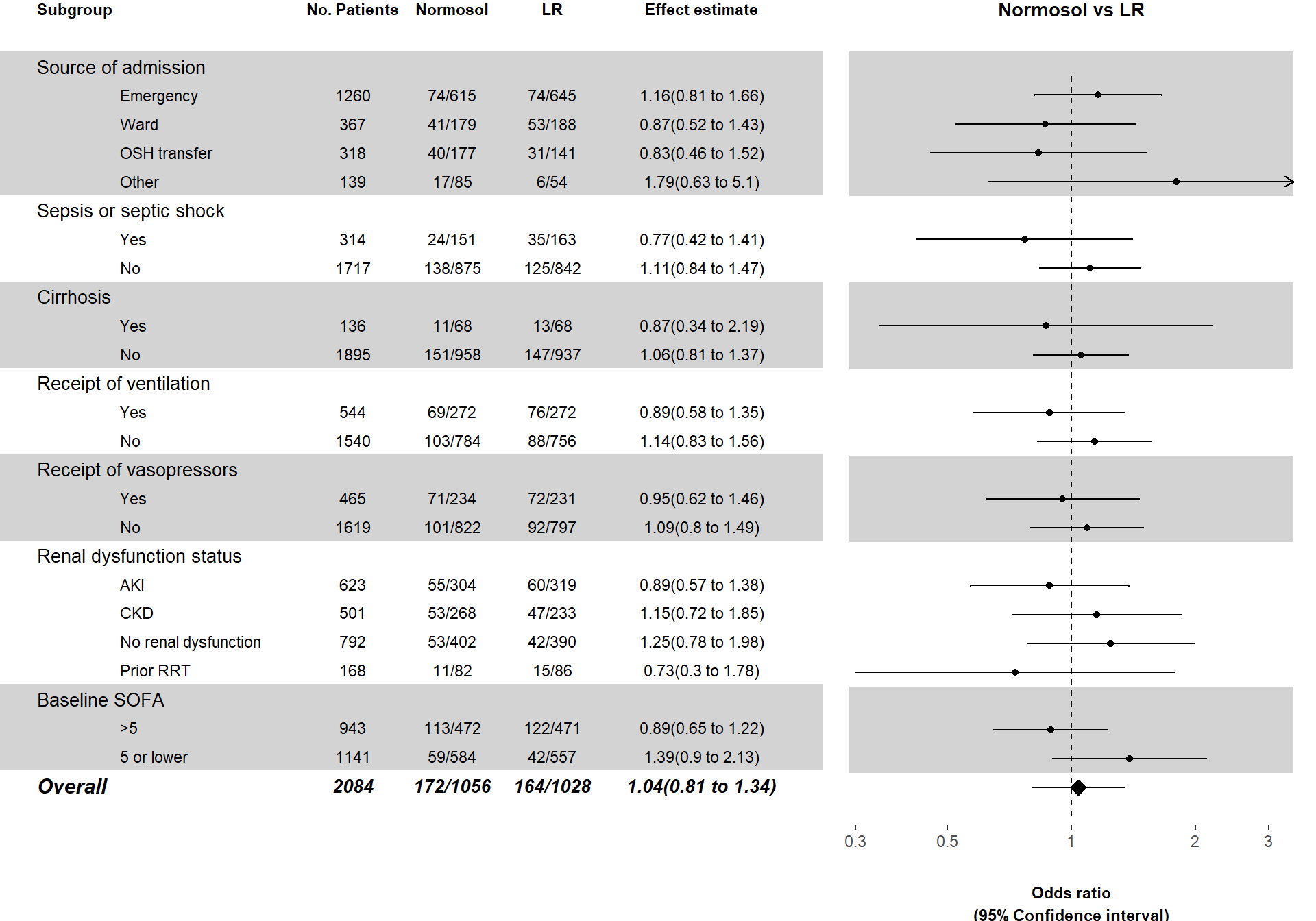


This figure displays the odds of 28-day in-hospital mortality in the lactated Ringer’s group (LR) and the Normosol-R. A value of 1.0 indicates similar odds of death in each group. Values less than 1.0 indicate lower odds of death with Normosol-R than with lactated Ringer’s. Value greater than 1.0 indicate lower odds of death with lactated Ringer’s than with Normosol-R. For sources of admission, Emergency refers to Emergency Department, Ward refers to a non-ICU floor within the hospital, and OSH transfer refers to a transfer from an outside hospital. AKI is acute kidney injury; CKD is chronic kidney disease, and RRT is renal replacement therapy. SOFA is the sequential organ failure assessment score.

### **SUPPLEMENTAL TABLES**

## **Table S1. First Daily Lab Values**

|  | **N** | **Normosol-R**  **(n = 1056)** | **Lactated Ringers**  **(n = 1028)** |
| --- | --- | --- | --- |
| Sodium (mEq/dL) |  |  |  |
| Day 1 | 1985 | 137 (134, 140) | 137 (134, 140) |
| Day 2 | 1923 | 138 (135, 140) | 138 (134, 140) |
| Day 3 | 1741 | 138 (135, 140) | 138 (135, 141) |
| Day 4 | 1508 | 138 (135, 141) | 138 (135, 141) |
| Day 5 | 1280 | 138 (135, 141) | 138 (136, 141) |
| Day 6 | 1110 | 138 (135, 141) | 138 (136, 141) |
| Day 7 | 939 | 138 (135, 141) | 138 (136, 141) |
| Day 8 | 786 | 138 (135, 141) | 138 (136, 141) |
| Day 9 | 660 | 138 (135, 140) | 138 (136, 141) |
| Day 10 | 586 | 138 (135, 140) | 138 (135, 141) |
| Day 11 | 511 | 138 (135, 141) | 138 (135, 141) |
| Day 12 | 449 | 138 (135, 141) | 138 (135, 141) |
| Day 13 | 400 | 138 (135, 141) | 138 (135, 140) |
| Day 14 | 350 | 138 (135, 141) | 138 (136, 141) |
|  |  |  |  |
| Potassium (mEq/dL) |  |  |  |
| Day 1 | 1986 | 4.2 (3.7, 4.8) | 4.2 (3.8, 4.9) |
| Day 2 | 1924 | 4.1 (3.7, 4.6) | 4.1 (3.7, 4.6) |
| Day 3 | 1738 | 4.0 (3.6, 4.4) | 4.0 (3.6, 4.4) |
| Day 4 | 1508 | 4.0 (3.6, 4.4) | 3.9 (3.6, 4.4) |
| Day 5 | 1279 | 4.0 (3.6, 4.4) | 3.9 (3.6, 4.3) |
| Day 6 | 1109 | 4.0 (3.6, 4.4) | 4.0 (3.6, 4.3) |
| Day 7 | 940 | 4.0 (3.6, 4.4) | 4.0 (3.7, 4.4) |
| Day 8 | 786 | 4.0 (3.6, 4.4) | 4.0 (3.7, 4.4) |
| Day 9 | 659 | 4.0 (3.6, 4.4) | 4.1 (3.7, 4.4) |
| Day 10 | 586 | 4.1 (3.7, 4.5) | 4.1 (3.7, 4.5) |
| Day 11 | 511 | 4.0 (3.7, 4.4) | 4.1 (3.7, 4.5) |
| Day 12 | 450 | 4.0 (3.7, 4.4) | 4.1 (3.7, 4.6) |
| Day 13 | 403 | 4.0 (3.7, 4.4) | 4.1 (3.7, 4.6) |
| Day 14 | 351 | 4.1 (3.7, 4.4) | 4.0 (3.6, 4.5) |
|  |  |  |  |
| Chloride (mEq/dL) |  |  |  |
| Day 1 | 1984 | 104 (99, 107) | 104 (99, 108) |
| Day 2 | 1922 | 105 (101, 108) | 105 (100, 109) |
| Day 3 | 1737 | 105 (100, 108) | 105 (100, 109) |
| Day 4 | 1508 | 104 (100, 108) | 105 (101, 109) |
| Day 5 | 1278 | 104 (100, 107) | 104 (100, 108) |
| Day 6 | 1107 | 104 (100, 107) | 104 (100, 108) |
| Day 7 | 937 | 104 (100, 107) | 105 (101, 108) |
| Day 8 | 785 | 103 (99, 107) | 105 (101, 108) |
| Day 9 | 659 | 103 (99, 107) | 104 (101, 108) |
| Day 10 | 585 | 103 (100, 107) | 105 (101, 108) |
| Day 11 | 510 | 104 (100, 107) | 105 (101, 108) |
| Day 12 | 448 | 104 (100, 107) | 105 (101, 108) |
| Day 13 | 399 | 104 (99, 107) | 104 (100, 107) |
| Day 14 | 350 | 104 (100, 107) | 103 (100, 107) |
|  |  |  |  |
| Bicarbonate (mEq/dL) |  |  |  |
| Day 1 | 1990 | 21 (17, 24) | 22 (18, 25) |
| Day 2 | 1926 | 22 (19, 25) | 22 (20, 26) |
| Day 3 | 1738 | 23 (20, 26) | 23 (20, 26) |
| Day 4 | 1511 | 24 (21, 27) | 24 (21, 27) |
| Day 5 | 1280 | 24 (21, 27) | 24 (21, 27) |
| Day 6 | 1108 | 24 (22, 28) | 24 (22, 27) |
| Day 7 | 937 | 24 (21, 28) | 24 (21, 28) |
| Day 8 | 784 | 25 (22, 28) | 24 (21, 27) |
| Day 9 | 659 | 25 (22, 29) | 25 (22, 27) |
| Day 10 | 584 | 25 (22, 28) | 24 (21, 28) |
| Day 11 | 511 | 25 (22, 28) | 24 (22, 27) |
| Day 12 | 447 | 25 (22, 28) | 24 (22, 27) |
| Day 13 | 398 | 25 (21, 29) | 24 (22, 27) |
| Day 14 | 350 | 25 (22, 29) | 25 (22, 28) |
|  |  |  |  |
| Creatinine (mg/dL) |  |  |  |
| Day 1 | 1983 | 1.2 (0.8, 2.3) | 1.2 (0.8, 2.4) |
| Day 2 | 1922 | 1.1 (0.8, 2.2) | 1.1 (0.8, 2.2) |
| Day 3 | 1738 | 1.0 (0.8, 2.2) | 1.1 (0.8, 2.0) |
| Day 4 | 1509 | 1.0 (0.7, 2.1) | 1.0 (0.8, 1.9) |
| Day 5 | 1280 | 1.0 (0.7, 2.2) | 1.0 (0.7, 1.8) |
| Day 6 | 1111 | 1.0 (0.7, 2.1) | 1.0 (0.7, 1.8) |
| Day 7 | 938 | 1.0 (0.7, 2.1) | 1.0 (0.7, 1.8) |
| Day 8 | 787 | 1.0 (0.7, 2.0) | 1.0 (0.7, 1.9) |
| Day 9 | 662 | 1.0 (0.7, 1.9) | 1.0 (0.7, 1.9) |
| Day 10 | 586 | 0.9 (0.7, 1.9) | 1.0 (0.8, 2.1) |
| Day 11 | 510 | 1.0 (0.7, 2.1) | 1.0 (0.7, 2.1) |
| Day 12 | 451 | 1.0 (0.7, 2.2) | 1.0 (0.7, 2.1) |
| Day 13 | 402 | 1.1 (0.7, 2.1) | 1.0 (0.7, 2.0) |
| Day 14 | 350 | 1.1 (0.7, 2.1) | 1.1 (0.8, 2.4) |
|  |  |  |  |
| Calcium (mEq/dL) |  |  |  |
| Day 1 | 1980 | 8.8 (8.1, 9.4) | 8.8 (8.2, 9.4) |
| Day 2 | 1921 | 8.5 (8.0, 9.0) | 8.6 (8.1, 9.1) |
| Day 3 | 1736 | 8.6 (8.1, 9.1) | 8.6 (8.1, 9.1) |
| Day 4 | 1508 | 8.7 (8.2, 9.1) | 8.6 (8.2, 9.2) |
| Day 5 | 1278 | 8.7 (8.2, 9.1) | 8.7 (8.2, 9.2) |
| Day 6 | 1108 | 8.7 (8.2, 9.2) | 8.7 (8.2, 9.2) |
| Day 7 | 935 | 8.7 (8.2, 9.2) | 8.7 (8.2, 9.2) |
| Day 8 | 785 | 8.7 (8.2, 9.3) | 8.7 (8.2, 9.2) |
| Day 9 | 659 | 8.7 (8.3, 9.2) | 8.7 (8.3, 9.2) |
| Day 10 | 585 | 8.7 (8.2, 9.2) | 8.7 (8.1, 9.2) |
| Day 11 | 509 | 8.7 (8.2, 9.3) | 8.6 (8.1, 9.2) |
| Day 12 | 448 | 8.7 (8.2, 9.2) | 8.6 (8.2, 9.1) |
| Day 13 | 398 | 8.7 (8.2, 9.3) | 8.7 (8.2, 9.2) |
| Day 14 | 349 | 8.8 (8.3, 9.2) | 8.7 (8.3, 9.1) |

Abbreviations: dL – deciliter; mEq – milliequivalent; mg - milligrams

All Data are presented as median [25^th^ percentile – 75^th^ percentile].

## **Table S2. Median Daily Cumulative Crystalloid Volumes**

|  | **Normosol-R**  **(n = 1056)** | **Lactated Ringers**  **(n = 1028)** |
| --- | --- | --- |
| Lactated Ringers |  |  |
| Day 1 | 0 (0, 0) | 0 (0, 700) |
| Day 2 | 0 (0, 0) | 245 (0, 1500) |
| Day 3 | 0 (0, 0) | 500 (0, 2000) |
| Day 4 | 0 (0, 0) | 500 (0, 2006) |
| Day 5 | 0 (0, 0) | 500 (0, 2212) |
| Day 6 | 0 (0, 0) | 500 (0, 2479) |
| Day 7 | 0 (0, 0) | 500 (0, 2500) |
| Day 8 | 0 (0, 0) | 600 (0, 2500) |
| Day 9 | 0 (0, 0) | 685 (0, 2500) |
| Day 10 | 0 (0, 0) | 695 (0, 2525) |
| Day 11 | 0 (0, 0) | 695 (0, 2565) |
| Day 12 | 0 (0, 0) | 728 (0, 2612) |
| Day 13 | 0 (0, 0) | 750 (0, 2700) |
| Day 14 | 0 (0, 0) | 750 (0, 2700) |
|  |  |  |
| Normosol-R |  |  |
| Day 1 | 0 (0, 944) | 0 (0, 0) |
| Day 2 | 250 (0, 1500) | 0 (0, 0) |
| Day 3 | 500 (0, 2000) | 0 (0, 0) |
| Day 4 | 500 (0, 2000) | 0 (0, 0) |
| Day 5 | 500 (0, 2134) | 0 (0, 0) |
| Day 6 | 500 (0, 2242) | 0 (0, 0) |
| Day 7 | 538 (0, 2262) | 0 (0, 0) |
| Day 8 | 605 (0, 2354) | 0 (0, 0) |
| Day 9 | 660 (0, 2400) | 0 (0, 0) |
| Day 10 | 725 (0, 2500) | 0 (0, 0) |
| Day 11 | 725 (0, 2500) | 0 (0, 0) |
| Day 12 | 750 (0, 2500) | 0 (0, 0) |
| Day 13 | 750 (0, 2530) | 0 (0, 0) |
| Day 14 | 750 (0, 2540) | 0 (0, 0) |

All data are presented as median [25^th^ percentile – 75^th^ percentile].

## **Table S3. Mean Daily Cumulative Crystalloid Volumes**

|  | **Normosol-R**  **(n = 1056)** | **Lactated Ringers**  **(n = 1028)** |
| --- | --- | --- |
| Lactated Ringers |  |  |
| Day 1 | 76 (362) | 555 (1123) |
| Day 2 | 139 (556) | 1114 (1827) |
| Day 3 | 183 (672) | 1403 (2270) |
| Day 4 | 230 (802) | 1576 (2544) |
| Day 5 | 279 (962) | 1713 (2799) |
| Day 6 | 326 (1083) | 1812 (3022) |
| Day 7 | 365 (1200) | 1890 (3214) |
| Day 8 | 411 (1323) | 1964 (3485) |
| Day 9 | 453 (1486) | 2018 (3676) |
| Day 10 | 489 (1574) | 2062 (3821) |
| Day 11 | 521 (1647) | 2091 (3879) |
| Day 12 | 549 (1718) | 2117 (3926) |
| Day 13 | 576 (1788) | 2134 (3944) |
| Day 14 | 602 (1849) | 2151 (3969) |
|  |  |  |
| Normosol-R |  |  |
| Day 1 | 634 (1373) | 45 (515) |
| Day 2 | 1279 (2499) | 100 (1050) |
| Day 3 | 1573 (3130) | 138 (1119) |
| Day 4 | 1731 (3475) | 184 (1225) |
| Day 5 | 1851 (3694) | 221 (1336) |
| Day 6 | 1937 (3937) | 252 (1425) |
| Day 7 | 2015 (4198) | 272 (1476) |
| Day 8 | 2074 (4461) | 306 (1590) |
| Day 9 | 2118 (4727) | 359 (1998) |
| Day 10 | 2162 (4931) | 397 (2373) |
| Day 11 | 2188 (4975) | 410 (2397) |
| Day 12 | 2220 (5018) | 421 (2406) |
| Day 13 | 2246 (5062) | 433 (2416) |
| Day 14 | 2263 (5100) | 442 (2431) |

All data are presented as mean (standard deviation).

## **Table S4. Daily 0.9% Sodium Chloride Volumes**

| Median (Interquartile Range) |  |  |
| --- | --- | --- |
| Day 1 | 0 (0, 0) | 0 (0, 0) |
| Day 2 | 0 (0, 0) | 0 (0, 0) |
| Day 3 | 0 (0, 0) | 0 (0, 0) |
| Day 4 | 0 (0, 0) | 0 (0, 0) |
| Day 5 | 0 (0, 0) | 0 (0, 0) |
| Day 6 | 0 (0, 0) | 0 (0, 0) |
| Day 7 | 0 (0, 0) | 0 (0, 0) |
| Day 8 | 0 (0, 0) | 0 (0, 0) |
| Day 9 | 0 (0, 0) | 0 (0, 0) |
| Day 10 | 0 (0, 0) | 0 (0, 0) |
| Day 11 | 0 (0, 0) | 0 (0, 0) |
| Day 12 | 0 (0, 0) | 0 (0, 0) |
| Day 13 | 0 (0, 0) | 0 (0, 0) |
| Day 14 | 0 (0, 0) | 0 (0, 0) |
|  |  |  |
| Mean (Standard Deviation) |  |  |
| Day 1 | 71 (301) | 101 (552) |
| Day 2 | 134 (762) | 160 (646) |
| Day 3 | 117 (655) | 153 (769) |
| Day 4 | 98 (565) | 155 (777) |
| Day 5 | 94 (535) | 130 (661) |
| Day 6 | 98 (610) | 154 (949) |
| Day 7 | 88 (546) | 144 (818) |
| Day 8 | 84 (514) | 97 (649) |
| Day 9 | 75 (532) | 86 (621) |
| Day 10 | 73 (577) | 74 (548) |
| Day 11 | 76 (612) | 77 (661) |
| Day 12 | 73 (601) | 71 (701) |
| Day 13 | 78 (643) | 55 (524) |
| Day 14 | 67 (586) | 67 (670) |

## **Table S5. Multivariate Modeling of the Primary Outcome**

|  | **Estimate (95% Confidence Interval)** |
| --- | --- |
| Treatment Group (Lactated Ringer’s: Normosol-R) | -0.12 (-0.61, 0.36) |
| Age (years) | 0.02 (0.01, 0.04) |
| Sex (Male:Female) | -0.22 (-0.71, 0.26) |
| Race |  |
| White:Black | 0.76 (0.15,1.37) |
| Other:Black | -0.53 (-1.83. 0.76) |
| Source of Admission |  |
| Operating Room:Emergency Room | 0.03 (-1.76, 1.81) |
| Outside Hospital Transfer:Emergency Room | 0.48 (-0.24, 1.21) |
| Other ICU:Emergency Room | 1.59 (0.37, 2.81) |
| Outpatient:Emergency Room | 0.66 (-2.94, 4.26) |
| Hospital Ward:Emergency Room | 0.56 (-0.10, 1.21) |
| Baseline receipt of mechanical ventilation | 0.68 (0.10, 1.26) |
| Baseline receipt of vasopressors | -1.70 (-2.34, -1.06) |
| Sepsis at baseline | -0.88 (-1.59, -0.17) |
| Cirrhosis at baseline | -2.40 (-3.36, -1.45) |

## **Table S6. Sensitivity Analyses of the Primary Outcome**

|  | **Mean Difference or Odds Ratio (95% Confidence Interval)** |
| --- | --- |
| **Original Adjusted Analysis** | |
| Plasma Bicarbonate Concentration | -0.12 (-0.61, 0.36) |
| **1) Restricted to only those enrolled outside of a washout period** | |
| Plasma Bicarbonate Concentration | -0.50 (-1.05, 0.05) |
| **2) Limited to the first medical ICU presentation in each encounter** | |
| Plasma Bicarbonate Concentration | -0.05 (-0.55, 0.45) |
| **Post-hoc analysis of plasma bicarbonate less than 20 mEq/dL or Death versus plasma bicarbonate concentration greater than 20 mEq/dL** |  |
| Less than 20 mEq/dL or Death:Greater than 20 mEq/dL | 1.14 (0.95, 1.36) |

## **Table S7. Effect Modification of the Primary Outcome**

| **Variable** | **N** | **Mean Difference**  **(95% Confidence Interval)** |
| --- | --- | --- |
| Source of admission |  |  |
| Emergency department | 1226 | -0.50 (-1.11, 0.12) |
| Hospital ward | 358 | 1.38 (0.23, 2.52) |
| Outside hospital transfer | 315 | -0.47 (-1.74, 0.81) |
| Other ICU | 84 | 1.18 (-1.26, 3.62) |
| Operating Room | 38 | -1.73 (-5.28, 1.83) |
| Outpatient | 12 | -1.77 (-9.25, 5.70) |
| Sepsis |  |  |
| Yes | 312 | -0.35 (-1.56, 0.85) |
| No | 1670 | -0.08 (-0.61, 0.45) |
| Cirrhosis |  |  |
| Yes | 136 | -0.02 (-1.85, 1.80) |
| No | 1846 | -0.13 (-0.63, 0.37) |
| Baseline mechanical ventilation |  |  |
| Yes | 535 | 0.85 (-0.09, 1.80) |
| No | 1498 | -0.47 (-1.03, 0.09) |
| Baseline vasopressors |  |  |
| Yes | 456 | 0.52 (-0.50, 1.54) |
| No | 1577 | -0.31 (-0.85, 0.24) |
| Renal Dysfunction at enrollment |  |  |
| No kidney injury | 762 | -0.18 (-0.96, 0.59) |
| Acute kidney injury | 607 | -0.60 (-1.47, 0.26) |
| Chronic kidney disease | 497 | 0.38 (-0.56, 1.32) |
| Prior kidney replacement therapy | 167 | -0.05 (-1.66, 1.57) |
| Sequential Organ Failure Assessment |  |  |
| Greater than 5 | 926 | 0.18 (-0.53, 0.88) |
| 5 or lower | 1107 | -0.40 (-1.05, 0.25) |
| Overall | 2033 | -0.12 (-0.61, 0.36) |

### **SUPPLEMENTAL REFERENCES**

1. Kidney Disease Improving Global Outcomes (KDIGO) Acute Kidney Injury Work Group. KDIGO clinical practice guideline for acute kidney injury. *Kidney Int Suppl*. 2012;2(1):1-138. doi:10.1038/kisup.2012.1

2. Semler MW, Self WH, Wanderer JP, et al. Balanced Crystalloids versus Saline in Critically Ill Adults. *N Engl J Med*. 2018;378(9):829-839. doi:10.1056/NEJMoa1711584

3. Závada J, Hoste E, Cartin-Ceba R, et al. A comparison of three methods to estimate baseline creatinine for RIFLE classification. *Nephrol Dial Transplant*. 2010;25(12):3911-3918. doi:10.1093/ndt/gfp766

4. Vincent JL, Moreno R, Takala J, et al. The SOFA (Sepsis-related Organ Failure Assessment) score to describe organ dysfunction/failure. On behalf of the Working Group on Sepsis-Related Problems of the European Society of Intensive Care Medicine. *Intensive Care Med*. 1996;22(7):707-710. doi:10.1007/BF01709751
